# Electron work function: an indicative parameter towards a novel material design methodology

**DOI:** 10.1038/s41598-021-90715-4

**Published:** 2021-06-02

**Authors:** Yuzhuo Luo, Yunqing Tang, Tsai-Fu Chung, Cheng-Ling Tai, Chih-Yuan Chen, Jer-Ren Yang, D. Y. Li

**Affiliations:** 1 Dept. of Chemical and Materials Engineering, University of Alberta, Edmonton, AB T6G 2H5 Canada; 2 Department of Materials Science & Engineering, National Taiwan University, Taipei, Taiwan, ROC; 3 Graduate Institute of Intellectual Property, National Taipei University of Technology, Taipei, Taiwan

**Keywords:** Engineering, Materials science

## Abstract

Electron work function (EWF) has demonstrated its great promise in materials analysis and design, particularly for single-phase materials, e.g., solute selection for optimal solid-solution strengthening. Such promise is attributed to the correlation of EWF with the atomic bonding and stability, which largely determines material properties. However, engineering materials generally consist of multiple phases. Whether or not the overall EWF of a complex multi-phase material can reflect its properties is unclear. Through investigation on the relationships among EWF, microstructure, mechanical and electrochemical properties of low-carbon steel samples with two-level microstructural inhomogeneity, we demonstrate that the overall EWF does carry the information on integrated electron behavior and overall properties of multiphase alloys. This study makes it achievable to develop “electronic metallurgy”—an electronic based novel alternative methodology for materials design.

## Introduction

Structural materials are designed mainly based on phase diagrams, various strengthening mechanisms, and thermodynamics, etc. The traditional metallurgy has made significant contribution to the success in production of various industrial materials. However, with the technological advance, materials are required for working under various harsh and extreme conditions. The traditional metallurgical principles do not always provide effective guidelines for material design or modification. A large number of trial-and-error tests are often needed when designing a new material or modifying existing materials to meet specific requirements. This costly process takes tremendous time and energy. It is thus highly desired that the material design can be more accurate and achieved based on more fundamental principles. As a matter of fact, mechanical properties of metallic materials are largely dependent on their electron states, which are related to the atomic bond strength and spatial atomic arrangements in crystalline lattices^1,2^. Significant effort has long been made to correlate properties of materials to their electron state based on quantum mechanic^3^, which is however complicated for material design, especially for structural materials which consist of various microstructural constituents. It is thus wished to have simple but fundamental parameters, which reflect the electron behavior of materials and can be used feasibly for material analysis and design.

**Figure 1:**
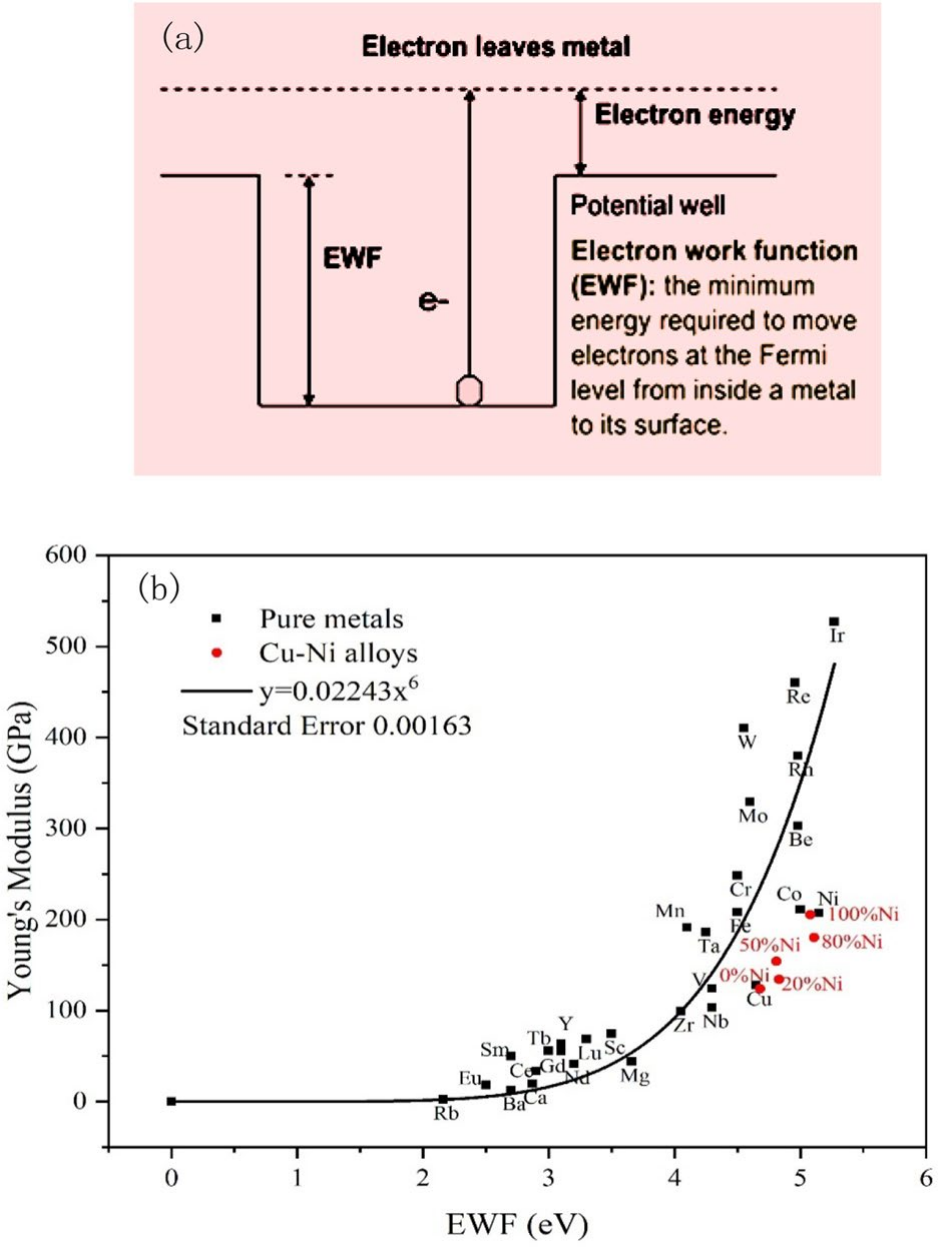
(**a**) Electron work function (EWF) is the minimum energy required to extract electrons at Fermi level from inside a metal to the position just out of its surface; (**b**) Correlation between EWF and Young’ modulus of pure metals (black dots) and Cu-Ni solid solution (red dots).

In recent years, considerable studies^4–6^ have demonstrated that electron work function, which is the minimum energy to move electrons at Fermi level inside a solid to its surface (see Fig. 1a), is a promising parameter to characterize materials and provide clues for material modification. EWF is related to the electron density, which influences the nuclei-electron and electron–electron interactions and thus determines the metallic bond strength^7^. Although EWF is a surface parameter, it is correlated with bulk properties because the electron density of the surface layer has a certain relationship with that of the bulk, although the surface lattice somewhat distorted and deviated from that of the bulk. As a result, the electron density-dependent EWF can inherently reflect the atomic bond strength that determines the bulk properties^8^. This parameter has already been demonstrated to be well correlated with the atomic bond strength^9^ and other atomic properties such as electronegativity and ionization energy^4,10,11^, which lays a theoretical foundation for utilizing EWF in material design. The dependence of many properties that are related to the atomic bond strength and stability, such as Young’s modulus^5^, yield strength and hardness^6^, fracture toughness^12,13^ and corrosion behavior^14^, on EWF has been proven theoretically and experimentally. For instance, Young’s modulus of metals is dependent on EWF^5^. Figure 1b illustrates collected experimental data on Young’s modulus and EWF of pure metals^15,16^, which fits well on a theoretical sixth power relationship^5^. In addition to the mechanical properties, EWF is also related with physical parameters of materials, such as surface energy^17^, adhesion^18,19^ and friction^20^, which play crucial roles in tribological and wearing processes.

Since EWF is related to the electron density^5^, the dependence of Young’s modulus on EWF is also applicable to multi-element solid solutions^21^. For instance, homogeneous Cu-Ni solid solutions show that their Young’s moduli and tribological properties have certain dependences on EWF^14,22,23^. The red dots in Fig. 1b illustrates Young’s moduli and EWFs of Cu-Ni alloys having different concentrations of Ni^22,24^, showing a similar trend as that for pure metals. The increase in %Ni brings in more electrons and thus raises EWF, leading to stronger atomic bonding and consequently higher barriers to any attempt to change the mechanical state. The enhanced atomic bonding correspondingly increases hardness and other hardness-dependent properties such as the wear resistance^23^. The corrosion resistance also increases with EWF due to the increased atomic bond stability that is related to the overall electrochemical stability^14^. Similar phenomena were observed when a limited amount of Ni was added to X70 steel as a solute element for strengthening purpose^25^. EWF can be used as an indicator to select effective solute elements for stronger solution-hardening effect. The solution hardening has two mechanisms related to mismatches in elastic modulus and atomic size between the solute and host elements^26,27^. Since these two factors mutually influence each other, how to select appropriate solute element for maximized hardening effectiveness was unclear. This issue has been well addressed with the help of EWF as a bridge, since EWF is related to the two mismatches and thus can be used as a guiding parameter to select solute element to achieve the maximum solution-strengthening effect^28^.

The previous studies on EWF related to material design are mainly conducted on metals and solid solutions without microstructural complexity. However, industrial materials are generally multiphase materials. Whether EWF can be used to guide design and modification of complex multiphase metallic materials remains a question, since it is unclear whether the overall EWF can reflect properties of a multiphase material with microstructural features at different levels. This is a main barrier for EWF to be used for structural materials design. Our preliminary experimental studies show that the overall or apparent EWF appears to be able to reflect the overall properties of two-phase materials. For instance, it was observed that adding Ni to X70 steel resulted in increases in both EWF and Young’s modulus of the steel. When a sufficient amount of Ni was added, EWF and Young’s modulus of the steel decreased due to the formation of a softer Fe_3_Ni phase^29^. However, in order to further confirm and understand the underlying mechanism, in-depth experimental and theoretical studies are needed.

In principle, when two phases in a material having different EWFs are in contact, electrons tend to move from the phase having a low EWF to that having a higher EWF, driven by the contact potential difference (CPD)^30^, until an electric dipole layer is established at the interface^18,31^. This process leads to redistribution of charges in the system, thus affecting its overall EWF, which may carry the information on the phase coupling and reflect the overall properties of the two-phase material. It was observed that EWF of Al-SiC nanocomposite increased as the fraction of SiC nanoparticles was increased although the spacing between SiC nanoparticles was around 6 times as large as the nanoparticle size^32^. This implies that the phase coupling is not only localized at the interphase boundary and the resultant effect could be delocalized. These studies indicate that interphase is a crucial factor and the effect of microstructure on EWF and overall properties could be connected to the phase coupling but the underlying mechanism needs to be clarified.

In this study, samples of a low-carbon steel (ASTM A109) having fine and coarse pearlites, respectively, were investigated to look into the effect of microstructure on EWF, mechanical properties, and the corrosion behavior. The fine and coarse pearlite microstructures were obtained by changing the rate of cooling after annealing. The treated samples showed two levels of microstructural differentiation. Samples experienced annealing and cooling in furnace (denoted as LCF) contain coarse pearlite and larger primary ferrite domains, while the samples experienced normalizing treatment (cooling in air; denoted as LCA) show fine pearlite and smaller primary ferrite domains. EWFs of the samples were measured using a Scanning Kelvin Probe and a Kelvin Probe Force Microscope (KPFM), respectively. Young’s moduli and hardness of the samples were measured for establishing relationships between EWF and the properties of the two-phase material, which is the main objective of the study. We have also conducted first-principles calculations to investigate how the cementite fraction and spatial arrangement influence the system’s work function. Results of the computational study are in agreement with the theoretical analysis and experimental observations.

## Methods

ASTM A109 carbon steel was used for the study, which contains 0.25%C, 0.60%Mn, Max. 0.04%P, Max. 0.60%Si, 0.04%S, 0.20%Cu, balanced by iron. Samples were treated in a tube furnace at 760 °C with argon atmosphere for 1 h. Half the samples were cooled in the furnace with 50 °C/h cooling rate (LCF). The other half of the samples were cooled in air (LCA). After the heat treatments, samples were cut and polished using SiC abrasive papers of 180, 320, 400, 800, 1200 grit successively, and then polished using 1 μm diamond slurry. A 2% nital solution was used as the etchant to remove a deformed layer and distinguish pearlite from ferrite, followed by ultrasonic cleaning in ethanol for 5 min and dried with a compressed air flow.

Optical micrographs (Mitutoyo Finescope, FS60), Scanning Electron Microscope (SEM, CamScan MV2300, UK) and Transmission Electron Microscope (TEM, a Hitachi H-7000) were used to analyze microstructures of the samples. Phase analysis was carried out using X-ray diffractometer (Rigaku Ultima IV with Cobalt tube at 38 kV and 38 mA) with a scanning range from 25° to 100° and the scanning speed was 2 deg/min. JADE 9.6 software was used to analyze the XRD spectra. Samples of 30 × 6 × 3 mm were cut for measuring their Young’s moduli using an acoustic instrument with RFDA basic software (IMCE company) for data analysis. Hardness was measured using an Indentec Hardness Testing Machine. At least five repeated measurements were carried out for each test. Electrochemical experiments were conducted at the room temperature using a Gamry electrochemical workstation. 3.5 wt% NaCl solution and 0.5 mol/L HCl acid solution were used, respectively, to measure corrosion resistances of the samples. The saturated calomel electrode (SCE) was reference electrode and Pt plate with 1 cm^2^ area was used as counter electrode. Scan rate was set to be 0.33 mV/s. Samples with dimensions of 10 × 10 × 5 mm were used for EWF analysis. A scanning Kelvin Probe (KP Technology, UK) with a gold tip was employed to measure overall work functions of the samples. Bruker Multimode Atomic Force Microscope8 (AFM) with PeakForce KPFM capability was used for EWF mapping using a Bruker magnetic probe, which can distinguish the potential difference between pearlite and ferrite domains. Interfacial area/volume ratio of pearlite to ferrite was analyzed by Image-Pro Plus 6.0.

Density functional theory^33^ (DFT) calculations were performed using Vienna ab-initio simulation package (VASP)^34–36^. The projector-augmented wave method was applied with the generalized gradient approximation (GGA) of Perdew-Burke-Ernzerhof (PBE)^37^. Cut-off energy of 450 eV and 2 × 2 × 1 k-point mesh^38^ was used for all calculations. The energy convergence condition for self-consistency calculations was 10^–5^ eV, and geometry relaxation tolerances were 10^–2^ eV/Å for force and 10^–5^ eV for energy. According to reported experimental observations^39^, Fe/Fe_3_C models were built with interfaces of $\left( {11\overline{2} } \right)_{Fe} //\left( {101} \right)_{{Fe_{3} C}}$ and $\left( {1\overline{1} 0} \right)_{Fe} //\left( {10\overline{1} } \right)_{{Fe_{3} C}}$, in which cementite was surrounded by the α-Fe matrix with different distribution densities. The boundary was periodic in plane perpendicular to interfaces. A vacuum layer of 15 Å was built above the surface, and the energy for an election moving from surface to vacuum was calculated as EWF of the system. The EWF was defined by the difference between the vacuum potential and the Fermi energy.

## Results

### Microstructure and phase analysis

**Figure 2:**
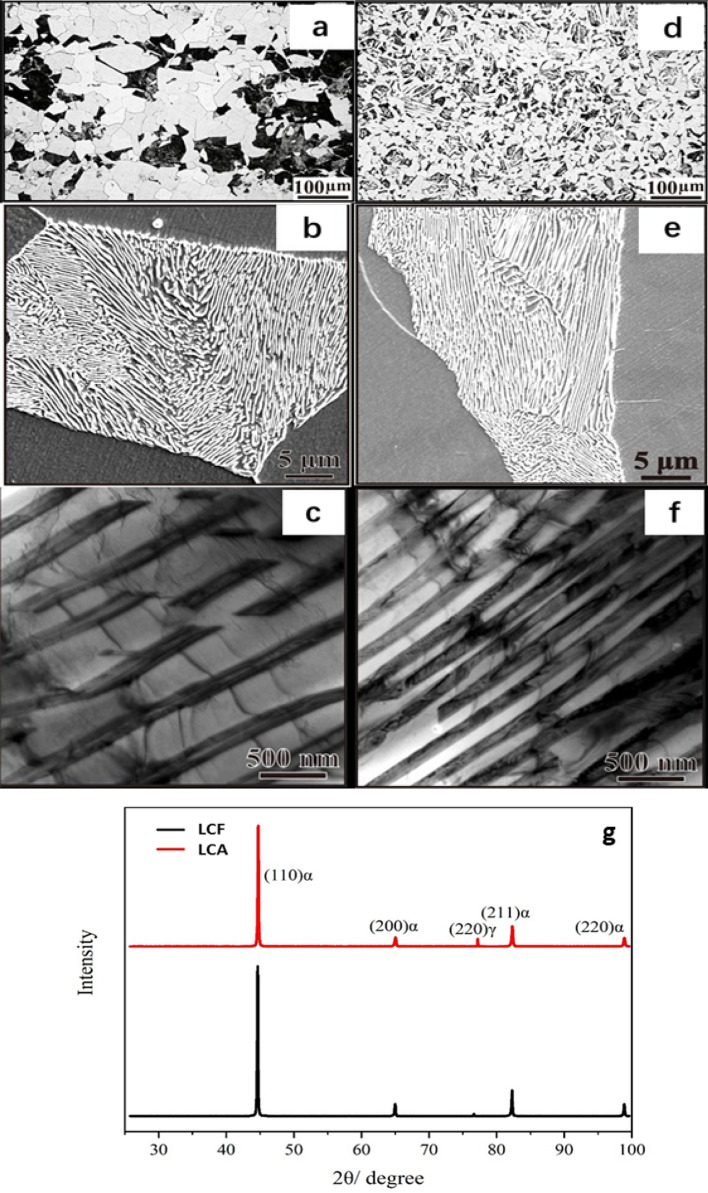
(**a**) An optical micrograph of LCF, dark regions are pearlites and white regions are the ferrite matrix; (**b**) A SEM image of LCF; (**c**) A TEM image of pearlite in LCF; (**d**) An optical micrograph of LCA; (**e**) A SEM image of LCA; (**f**) A TEM image of pearlite in LCA; (**g**) XRD patterns of LCF and LCA samples.

Representative optical, SEM and TEM images of the low-carbon steel are presented in Fig. 2a–f. A coarse pearlite microstructure of the LCF sample having its average grain size of about 50 μm is illustrated in Fig. 2a–c. At a lower cooling rate when cooled in furnace, austenite has more time to transform into coarse pearlite^40^. In this sample, the primary ferrite domains are larger. In contrast, the LCA sample, which was cooled in air after annealing, shows fine pearlite (average grain size is around 20 μm) with more densely distributed cementite in the pearlite domains, as Fig. 2d–f illustrate. With similar volume fractions of pearlite, the LCA sample shows a larger total interfacial area between pearlite and the ferrite matrix. As the TEM images in Fig. 2c,f illustrate, LCA sample has finer and denser plate-like cementite, compared to the LCF, corresponding to a larger total interfacial area between ferrite and cementite.

In general, a finer microstructure is harder than a coarse microstructure of the same material. In order to make sure that there are no unexpected changes in phase constituents, XRD analysis was conducted. Figure 2g shows XRD patterns of the LCF and LCA samples. Comparing with standard powder diffraction patterns, Fe (01-087-0721) and Fe_3_C (03-065-2412) are present in samples without existence of other phases. It should be indicated that the main peaks of cementite are around 45 degrees, overlapping with that of (110)_α_, and others are too weak to be detected. Based on the microstructure observations and XRD analysis, the furnace cooling and air cooling did not result in changes in phase constituents but affected the degree of microstructural coarsening.

### Dependence of mechanical properties on EWF

**Figure 3:**
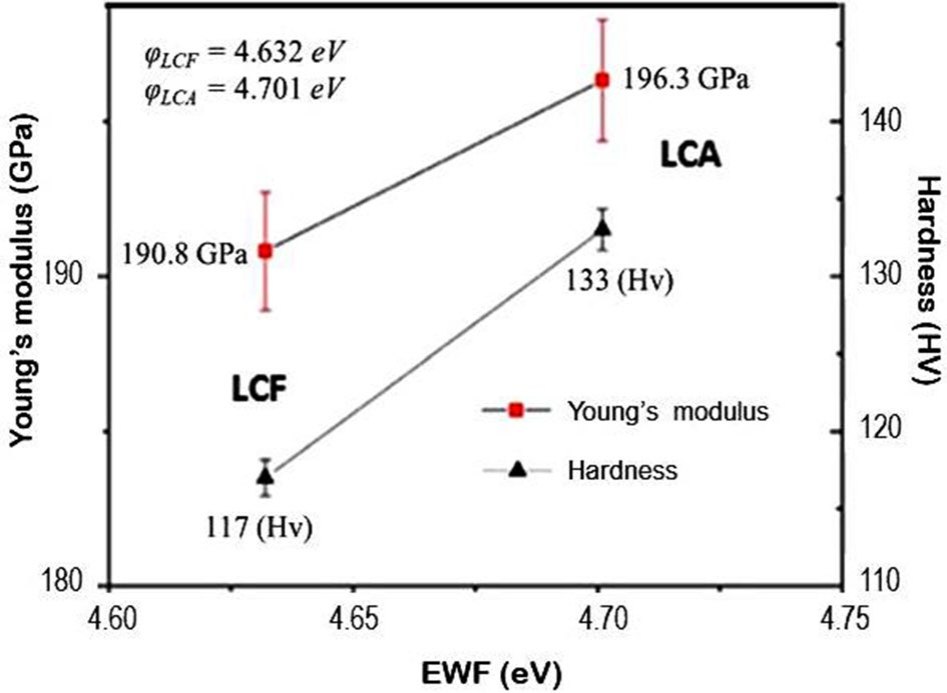
Relationship between mechanical property and overall EWF.

Young’s moduli, hardness values and overall work functions of the low-carbon steel samples were measured. The mechanical properties versus EWF are presented in Fig. 3. As shown, both Young’s modulus and hardness of the LCA sample show higher values, corresponding to its higher EWF. The results are consistent with previous observations that the trend of changes in mechanical property is similar to that of corresponding changes in electron work function^25,29,32,41^. The value of overall EWF represents the stability of electrons in the material system, which is related to the average atomic bond strength. The LCA sample has finer pearlite microstructure with a larger total interfacial area, which may lead to stronger electron redistribution. A finer microstructure generates stronger confinement to atoms in the system, which can be reflected by a higher EWF. Young’s modulus is intrinsically dependent on the atomic bond strength. The higher Young’s modulus of the LCA sample and correspondingly higher EWF imply that the overall mechanical strength and the overall or apparent EWF of a two-phase alloy should be correlated in a certain way. Electrons must be redistributed in order to reflect the changes in the overall properties that integrate contributions from various phases with certain microstructural features. Such electron redistribution leads to the development of a certain relationship between the mechanical properties and EWF, which may be similar to those for pure metals and homogeneous solid solutions^5,22^. As shown in Fig. 3, the change in hardness is similar to that of Young’s modulus. Although hardness is less intrinsic, a higher EWF leads to stronger atomic bonding and thus higher resistance to plastic deformation involving dislocation generation and movement^6,28^. As a result, both hardness and Young’s modulus show higher values with correspondingly higher overall EWF, indicating the dependence of the properties of the two-phase steel on its EWF.

### Relationship between the corrosion behavior and EWF

**Figure 4:**
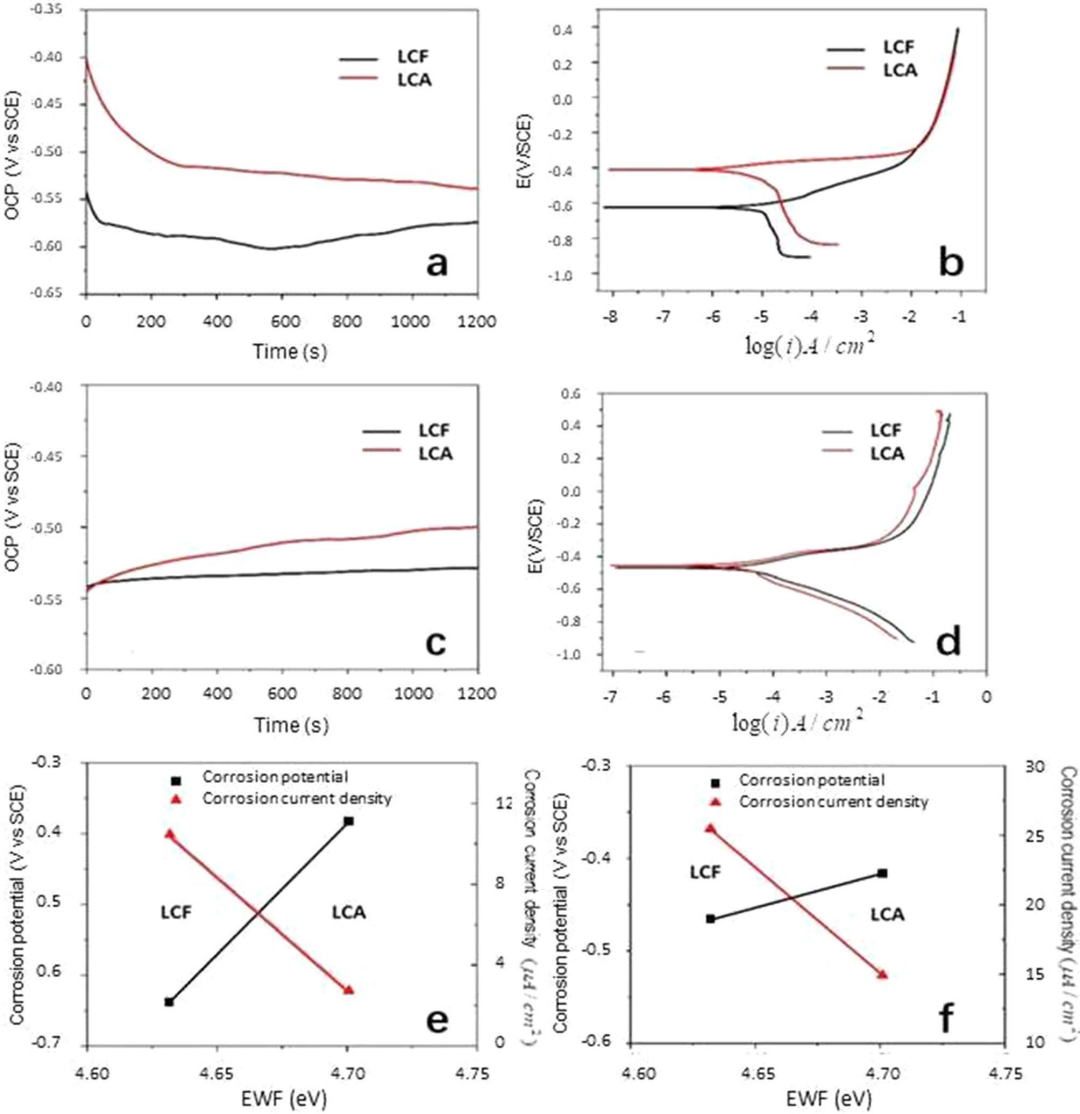
(**a**) Open circuit potentials and (**b**) polarization curves of samples LCF and LCA in 3.5 wt% NaCl solution; (**c**) Open circuit potentials and (**d**) polarization curves of samples LCF and LCA in 0.5 mol/L HCl solution; Relationship between EWF and corrosion potential, corrosion current density of the samples in 3.5 wt% NaCl solution (**e**) and 0.5 mol/L HCl solution (**f**).

The corrosion behavior of a material is related to its electrochemical stability, which is determined by the stability of atomic bonds. Thus, higher EWF corresponds to higher intrinsic corrosion resistance^14^. Here, the intrinsic corrosion resistance does not include influences from surface adsorption and oxidation, e.g., the formation of passive films. Or in other words, it refers to the resistance to material dissolution in a corrosive environment. Corrosion resistance can be reflected by two parameters, one is the corrosion rate (CR) which is directly related to the reaction kinetics and the other is the corrosion potential (E_corr_) which is a measure of the corrosion tendency of a material for starting corrosion. Based on Faraday’s law^42^, the corrosion rate is represented as:

1 $$Corrosion \, rate = \frac{{k \times j_{corr} \times EW}}{\rho }$$

where k is 0.00327 mm × g/(μA × cm × yr) for 3.5 wt% NaCl salt solution, j_corr_ is the corrosion current density (μA/cm^2^), EW is the equivalent weight of mild steel which is estimated to be 28.25. $\uprho$ is the density of steel equal to 7.86 g/cm^3,42^. In this study, a 3.5 wt% NaCl salt solution and a dilute HCL solution are used to investigate the corrosion behaviors of the LCF and LCA samples. In the NaCl solution, Cl^−^ plays a main role in corroding the steel, which is mainly influenced by pitting and intergranular corrosion effect^43^. Figure 4a illustrates the open circuit potentials (OCP) of the two samples against time. As shown, the LCA sample has a higher OCP with less tendency of being corroded. Polarization curves of two samples are illustrated in Fig. 4b. Using extrapolation method, the corrosion potential and corrosion current density can be determined based on the polarization curve. Results of the measurement are given in Table 1. As shown, the corrosion current density of LCA is lower than that of LCF, indicating that the LCA sample has a lower kinetic rate of corrosion in the salty solution. For more information, corrosion behaviors of the two samples in a 0.5 M HCl acid solution were also analyzed, in which carbon steel generally dissolves without formation of a surface film that complicates the corrosion process. The dilute HCL acidic solution is usually used as an aggressive corrosion medium in industrial processes such as pickling and etching^44^. Figure 4c,d show OCPs and polarization curves of the two samples in the acidic solution, respectively. The LCA sample still shows lower corrosion tendency, compared to that of the LCF sample, while their polarization curves are closer. Table 1 provides values of corrosion potential, corrosion current (I_corr_), corrosion current density and corrosion rate of the two samples. As shown, the corrosion current density of LCA is lower than that of LCF but the percentage difference is not as large as that in the NaCl solution, since the carbon steel is more prone to acidic solution.

**Table 1:** Corrosion potentials (E_corr_), corrosion currents (I_corr_), corrosion current densities (j_corr_) and corrosion rates (CR) of LCF and LCA samples in the two different solutions.

Solution	Sample	E_corr_/V	I_corr_/μA	j_corr_/(μA/cm^2^)	CR/(mm/yr)
3.5 wt% NaCl	LCF	− 0.638	21.21	10.45	0.123
	LCA	− 0.382	5.25	2.74	0.032
0.5 mol/L HCl	LCF	− 0.466	30.01	25.47	0.299
	LCA	− 0.416	28.69	14.96	0.176

Based on the measured corrosion parameters, the relationship between EWF and the corrosion resistance is clearly shown in Fig. 4e,f. With higher overall EWF that corresponds to a more stable state of electrons, the LCA sample shows higher resistance to corrosion (lower CR and higher E_corr_), compared with LCF sample in both the acidic and salty solutions.

## An interphase model and evaluation

The experimental observations have shown clear correlation between the EWF and both the mechanical and electrochemical properties of the carbon steel with different microstructural features. Such correlation suggests that EWF does reflect the overall properties of a two-phase or multiphase material, which are integrated from corresponding properties of individual phases that are EWF-dependent and affected by their microstructural characteristics. EWF is thus a promising parameter for analyzing materials and helping material design through appropriate microstructural arrangement. To achieve these, we need to establish a theoretical connection between the measured or apparent EWF and those of individual phases, and understand the underlying mechanism.

### EWF of pearlite—a microconstituent consisting of cementite and ferrite

**Figure 5:**
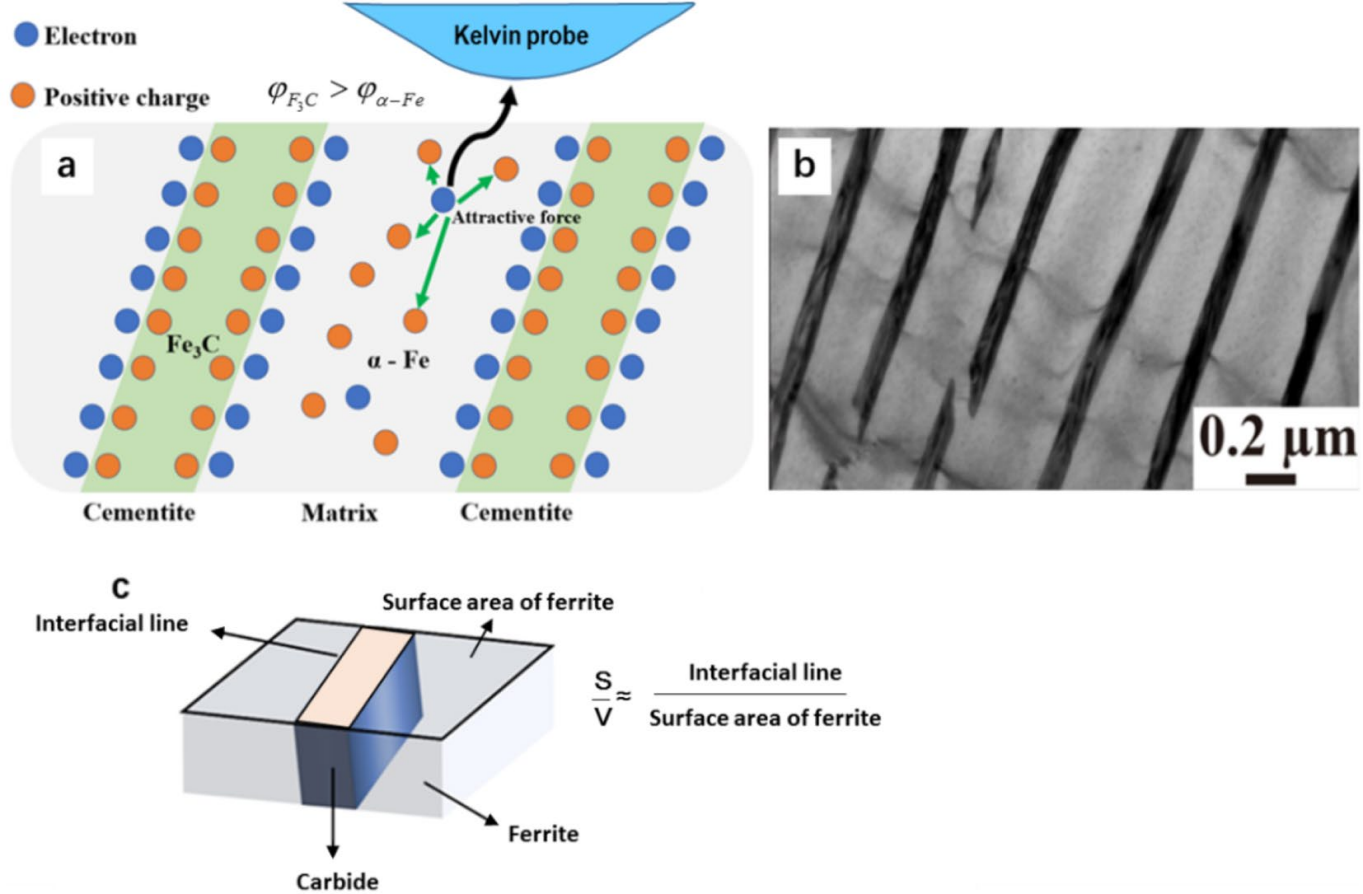
(**a**) Schematic of the charge-compensation model: electrons move towards the Fe/Fe_3_C interface, building a dipole layer to stop further charge accumulation. The ferrite region becomes electron-depleted, (**b**) A TEM image of pearlite consisting ferrite and cementite (dark), (**c**) 3D schematic of pearlite.

Let’s look at the pearlite first, which consists of two phases, ferrite ($\alpha - Fe$) and cementite ($Fe_{3} C$). When two different phases are in contact, electrons move from the phase having a lower EWF to that with a high EWF, driven by a contact potential difference^30,45^. In the present case, electrons in ferrite tend to move towards adjacent cementite that has a higher potential or EWF until a dipole layer is established at the Fe/Fe_3_C interface. As shown in Fig. 5, electrons are accumulated at the interface, which would build an electric field within the interface region as the charge accumulation, generating an opposite electrostatic force to balance the driving force resulting from the contact potential difference^31,45^. As electrons move towards the interfacial area, electrons are depleted in the ferrite region.

Such charge relocation leads to a positively charged ferrite region, from which electrons would have increased difficulty to escape when under an external electrical field during EWF measurement. Equivalently, the measured EWF would be higher. This mechanism may be revealed using a charge-compensation approach as described below.

As electrons in the ferrite phase are driven towards the Fe/Fe_3_C interface, equivalently the ferrite phase has a change in density of free electrons, $\Delta {\uprho }_{\mathrm{e}-}$, which is expressed as:

2 $$\Delta \rho_{{e^{ - } }} = \frac{S\sigma }{V}$$

where S represents the total carbide/ferrite interfacial area and $V$ is the total volume of the ferrite phase, $\upsigma$ is the interfacial electric density. The initial work function of the ferrite phase is determined by its initial electron density (${\uprho }_{\mathrm{e}-}$)^5^:

3 $$\varphi_{initial} = \alpha^{\prime}\rho_{{e^{ - } }}^{{{\raise0.5ex\hbox{$\scriptstyle 1$} \kern-0.1em/\kern-0.15em \lower0.25ex\hbox{$\scriptstyle 6$}}}}$$

where $\alpha^{\prime}$ is a material constant. As cementite with a higher work function is embedded in the ferrite matrix, the apparent or measured EWF may have the following change^31^:

4 $$\Delta \varphi = \frac{d\varphi }{{dx}} = \alpha^{\prime}\frac{1}{6}\rho_{{e^{ - } }}^{{ - \tfrac{5}{6}}} \Delta \rho_{{e^{ - } }}$$

The resultant percentage increase in work function is thus represented as a function of the interfacial and volume parameters:

5 $$\frac{\Delta \varphi }{{\varphi_{initial} }} = \frac{{\Delta \rho_{{e^{ - } }} }}{{6\rho_{{e^{ - } }} }} = \frac{S\sigma }{{6V\rho_{{e^{ - } }} }}$$

**Figure 6:**
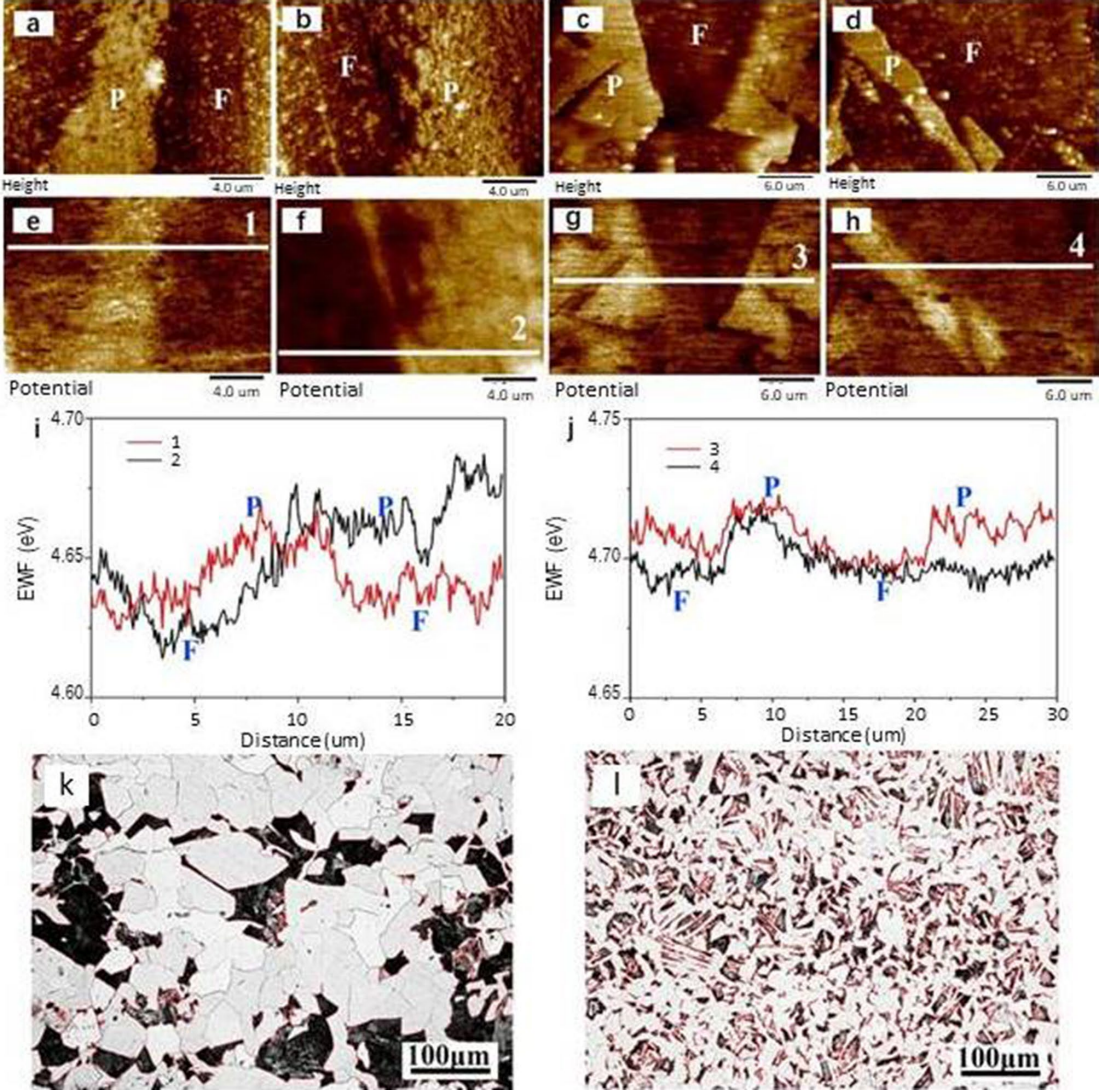
**(a-d)** Topographical maps of LCF (**a**,**b**) and LCA (**c**,**d**), P represents pearlite and F represents ferrite in the images; (**e**–**h**) corresponding potential maps of LCF (**e**,**f**) and LCA (**g**,**h**); (**i**) line profile of potential change [along lines 1 and 2 in (**e**) and (**f**) respectively] in LCF, (**j**) line profile of potential change [along lines 3 and 4 in (**g**) and (**h**), respectively] in LCA; (**k**) optical micrographs of LCF with red interfacial lines; (**l**) optical micrographs of LCA with red interfacial lines.

Since work function is the energy required to attract an electron from a metal surface layer, the S/V ratio is estimated using the ratio of the total length of interfacial line to the surface area of ferrite. With a higher S/V ratio, corresponding to a finer microstructure, the measured EWF would be increased with correspondingly changed mechanical and electrochemical properties. When the steel is cooled at a higher cooling rate, the thickness of plate-cementite in pearlite becomes smaller with a larger S/V ratio. It is expected that the pearlite in the LCA sample would have a higher EWF than that of pearlite in the furnace-cooled sample LCF in which the pearlite is coarser (see Fig. 2). In order to confirm this, EWFs of pearlite domains in LCA and LCF samples were analyzed through AFM mapping. Figure 6a–d show representative topographical maps of LCF and LCA samples, respectively. Corresponding potential maps of the samples are illustrated in Fig. 6e–h. The light area represents pearlite (P) and dark one represents ferrite (F). Figure 6i,j show local potential difference between LCF and LCA measured along the white lines in Fig. 6e–h. Statistical results confirm that pearlite shows higher potential compared with ferrite (see Table 2, which gives the measured EWFs of perlite domains in LCF and LCA samples). As shown, EWF of the pearlite in LCA sample is higher than that in LCF sample, consistent with the theoretical analysis.

**Table 2:** Statistical local work function in LCF and LCA samples.

Local work function (eV)	Pearlite	Ferrite
LCF	4.664 ± 0.0114	4.629 ± 0.0072
LCA	4.715 ± 0.0043	4.688 ± 0.0018

### EWF of the steel—an alloy system consisting of pearlite and ferrite

For the air-cooled and furnace-cooled samples, their microstructures are different at two levels. The microstructural difference at level one refers to the difference in size and spacing of cementite in pearlite between sample LCF and sample LCA as shown in Fig. 2c,f and discussed in the previous section. The microstructural difference at level two refers to the difference in the sizes of pearlite and ferrite domains between the samples as Figs. 2a,d or 6k,l illustrate. Since pearlite has a higher EWF than ferrite as Fig. 6i,j illustrate, a dipole layer would form at the pearlite/ferrite interface as well. Thus, EWF of the samples would also be influenced by microstructure at this level involving the size of pearlite domains and the spacing between adjacent pearlite domains.

For the pearlite/ferrite interface, the interfacial charge density $\upsigma$ should be defined. Based on Poisson’s equation on electrostatics^46^, contact potential V at interface can be related to the interfacial electric density $\rho_{{\text{int}}}$(Coulomb):

6 $$\nabla^{2} V = \frac{{d^{2} V}}{{dx^{2} }} = - \frac{{\rho_{{\text{int}}} }}{{\varepsilon_{ \circ } }}$$

The electric density of the dipole layer, $\upsigma$, is thus equal to

7 $$\rho_{{\text{int}}} = \sigma e$$

8 $$\Delta \varphi = \varphi (pearlite) - \varphi (ferrite)$$

We have

9 $$\frac{{d^{2} (\Delta \varphi )}}{{dx^{2} }} = \frac{{\sigma e^{2} }}{{\varepsilon_{ \circ } }}$$

So interfacial charge density can be expressed as:

10 $$\sigma = \frac{\varphi (pearlite) - \varphi (ferrite)}{{x^{2} e^{2} }}$$

where x is the width of the dipole layer at pearlite/ferrite interface. According to theoretical calculation, the typical width of p–n junction depletion layer is 50–1000 Å^47^. To simplify the analysis without losing physical significance, we assume x = 50 Å for the metallic pair of ferrite and pearlite. The calculated interfacial charge densities are given in Table 3. The magnitude of change in work function is affected by the S/V ratio and σ as Eq. (5) expresses; here S is the total interfacial area between pearlite and ferrite, and V is the total volume of ferrite outside pearlite i.e. primary ferrite. The S/V ratio is estimated by the ratio of total pearlite/ferrite interfacial line to the total surface area of primary ferrite. Figure 6k,l give micrographs of LCF and LCA processed by Image-pro Plus. Interface between pearlite and ferrite is drawn by red line. As shown in Table 3, S/V ratio increases significantly in the LCA sample, which is more than three times as high as that of LCF. This makes a main contribution to the charge-compensation effect, leading to increased overall electronic stability. Free electron density ${\uprho }_{\mathrm{e}-}$ is determined by density parameter r_s_ (Fe: 1.04 Å)^48^, so initial matrix’s free electron density is set to be 2.12 × 10^29^ electrons/m^3^. The increasing percentage of apparent work function is given in Table 3. As shown, sample LCA has its EWF higher than that of LCF, resulting from its fine and dense pearlite configurations, corresponding to better mechanical performance. If the initial work function of iron is set as 4.5 eV^48^, the theoretical work function can also be provided, which shows a good fit with experimental data. This microstructure—EWF—property model works well for the steel with the microstructural features.

**Table 3:** Detailed data (e.g., interfacial charge density, S/V, etc.) used in model calculations.

Sample	LCF	LCA
△φ (eV)	0.035	0.027
σ (10^23^/m^2^)	1.55	1.19
Percentage of pearlite (%)	0.2846	0.2445
Interfacial line (μm)	12,219	41,719
Matrix area (μm^2^)	164,899	174,143
S/V (10^6^/m)	0.074	0.240
△φ/φ_initial_ (%)	0.90	2.25
φ_*Predicted*_ (eV)	4.541^a^	4.601^a^
φ_*Measured*_ (eV)	4.632	4.701

^a^The work function of iron is 4.5 eVs^48^.

### Model confirmed by first-principle calculations

**Figure 7:**
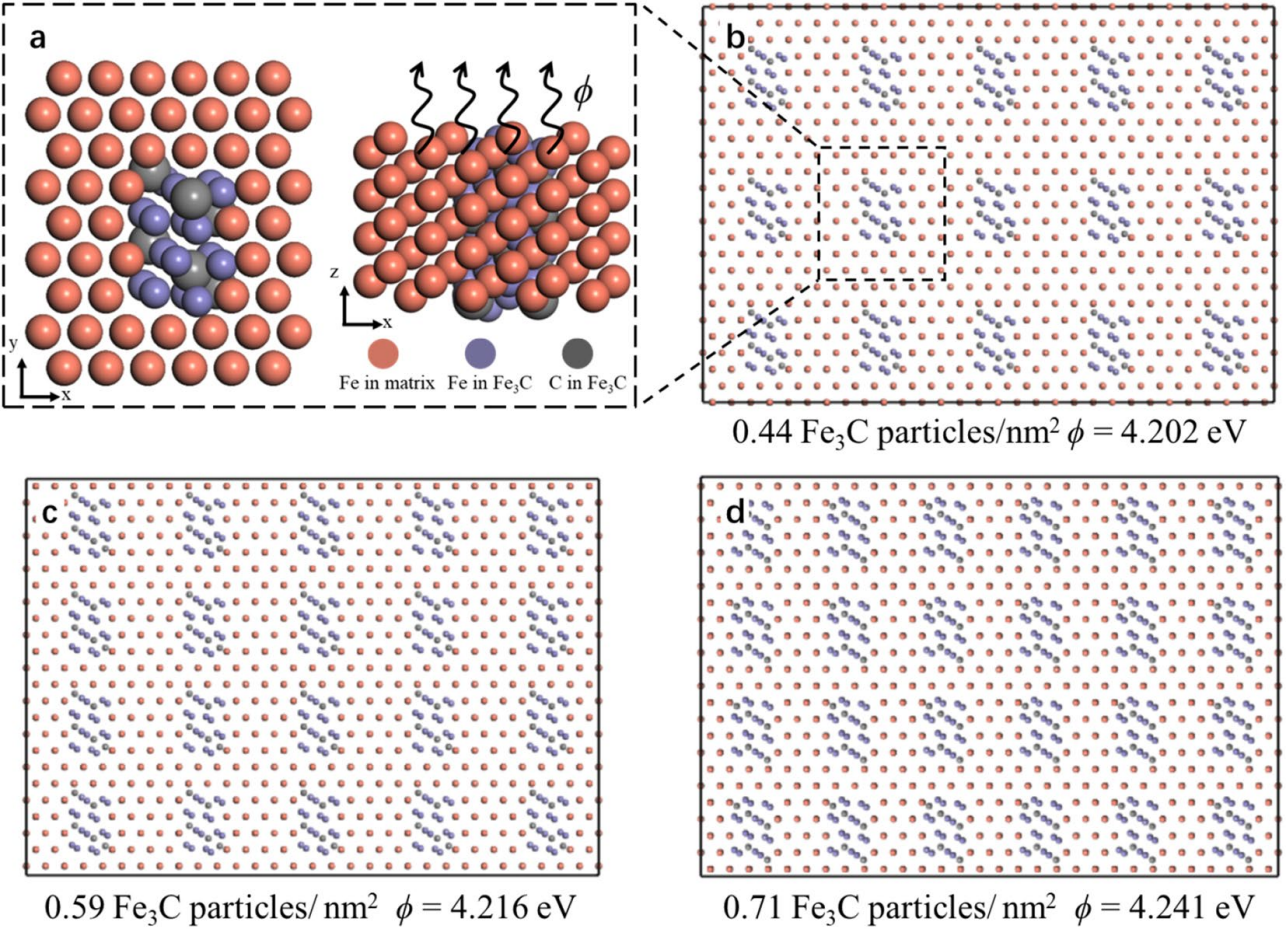
Schematic interface between Fe and Fe_3_C with three different configurations. (**a**) Fe/Fe_3_C interface influences the escape of electrons at fermi level from inside the system consisting of cementite and α-Fe matrix (ferrite) to vacuum. (**b**–**d**) Three systems with different distribution densities of cementite in the α-Fe matrix and corresponding work functions.

The charge-compensation model shows that the interfacial area between phases induces redistribution of valence electrons and thus influences the apparent overall EWF of samples. We performed first-principle calculations to further confirm this phenomenon. Though the model systems shown in Fig. 7 for the first-principles analysis are smaller than the real systems, the main purpose of the calculations is to verify the theoretical consideration rather than providing accurate values for precise quantitative comparison with experimentally measured values. The influence of the ferrite/cementite interfacial coupling on the EWF of the system is well revealed by the calculations. As shown, EWF of the system increases as the total ferrite/cementite interfacial area increases, resulting from an increase in the fraction of cementite having the same domain size in the system.

Interfaces of $\left( {11\overline{2} } \right)_{Fe} //\left( {101} \right)_{{Fe_{3} C}}$ and $\left( {1\overline{1} 0} \right)_{Fe} //\left( {10\overline{1} } \right)_{{Fe_{3} C}}$ were built as they were observed in experiments^39,49^. In the built system as shown in Fig. 7, the exposed surfaces of ferrite and cementite phases are $\left( {111} \right)_{Fe}$ and $\left( {010} \right)_{{Fe_{3} C}}$, respectively. The interfaces in the constructed systems (Fig. 7) are perpendicular to the system’s surface, which are $\left( {111} \right)_{Fe}$ and $\left( {010} \right)_{{Fe_{3} C}}$, respectively. As EWF of ferrite is lower than that of cementite, computational EWFs of the system having Fe/Fe_3_C interfaces should be closer to EWF of $\left( {111} \right)_{Fe}$ surface, which is lower than that of the most stable ferrite $\left( {110} \right)_{Fe}$ surface. Previous calculations show that the work function of Fe (111) plane is in the range of 3.84–4.25 eV^50–53^. EWF of $\left( {111} \right)_{Fe}$ surface obtained from our calculation with the density functional theory is 4.17 eV. EWFs of systems consisting ferrite and cementite having different distribution densities (0.44, 0.59 and 0.71 particles/nm^2^) were calculated. As illustrated in Fig. 7, with increasing the distribution density of Fe_3_C in the ferrite matrix, the interfacial area between Fe/Fe_3_C increases, leading to enhanced electron confinement in the iron matrix, corresponding to elevated apparent EWF of the system. Results of the calculations are consistent with the mechanism elucidated by the charge-compensation model.

## Conclusions

Electron work function is inherently correlated to the metallic bond strength and stability of metals, thus largely affecting their mechanical and electrochemical properties. However, whether or not the overall or apparent EWF can reflect overall properties of multiphase alloys remains a question. This is a main barrier to the application of EWF in guiding material design or modification. In this study, we investigated the effect of microstructure on EWF and properties of ASTM A109 carbon steel samples respectively cooled in furnace and in air after annealing, which showed difference in microstructural inhomogeneity at two levels between samples LCF and LCA: level 1 (refer to pearlite that consists of Fe_3_C and ferrite)—differences in the size of Fe_3_C precipitates in pearlite and the spacing between adjacent Fe_3_C domains, and level 2 (refer to the steel that consists of pearlite and ferrite)—differences in size of pearlite domain and the spacing between adjacent pearlite domains. The properties under study include Young’s modulus, hardness and corrosion behavior. We have observed the following phenomena and elucidated underlying mechanisms.

1. As demonstrated, the sample cooled in air (LCA) has its pearlite containing thinner cementite plates embedded in ferrite with a smaller spacing between adjacent cementite plates. The pearlite in the sample cooled in air (LCA) shows a higher EWF, compared to that in the furnace-cooled sample (LCF).
2. The LCA sample has a finer microstructure with smaller pearlite (P) and ferrite (F) domains and its total P/F interfacial area is considerably larger than that in LCF, resulting in further increased EWF. The normalized sample has a higher overall EWF than the furnace-cooled sample.
3. When two microconstituents having different EWFs are in contact, electrons move from the low-EWF one to that having a higher EWF until the formed dipole layer at the interface is sufficiently strong to stop the electron migration. Equivalently the low-EWF microconstituent becomes positively charged, leading to enhanced confinement to electrons and thus elevated overall EWF.
4. Higher overall EWF corresponds to stronger overall confinement to electrons, resulting in stronger atomic bonding and stability, corresponding to higher mechanical strength and larger resistance to corrosion.

Up to this point, we demonstrate that the overall or apparent EWF does carry the information on integrated electron behavior and overall properties of multiphase alloys. Establishment of such relationships is a crucial step towards the design of structural materials on a feasible electronic base or through “electronic metallurgy” as a complementary or alternative methodology.

## Data Availability

Data in this article are self-containing.
